# Abnormal lipopolysaccharide binding protein as marker of gastrointestinal inflammation in Parkinson disease

**DOI:** 10.3389/fnins.2015.00306

**Published:** 2015-09-01

**Authors:** Gian D. Pal, Maliha Shaikh, Christopher B. Forsyth, Bichun Ouyang, Ali Keshavarzian, Kathleen M. Shannon

**Affiliations:** ^1^Department of Neurological Sciences, Rush University Medical CenterChicago, IL, USA; ^2^Department of Internal Medicine, Section of Gastroenterology, Rush University Medical CenterChicago, IL, USA

**Keywords:** lipopolysaccharide, gastrointestinal, biomarker, Parkinson disease

## Abstract

**Objective:** An inflammation-driven model of PD has been proposed based on the endotoxin lipopolysaccaride (LPS), a potential source of inflammation in the gastrointestinal system linked to neurotoxicity. Systemic exposure to bacterial endotoxin (LPS) can be determined by measuring plasma LPS binding protein (LBP). We aimed to evaluate whether lipopolysaccharide binding protein (LBP) can be used to distinguish PD subjects from control subjects and to assess whether LBP levels correlate with PD disease severity.

**Methods:** We measured plasma LBP (ng/ml) using an ELISA kit in 94 PD subjects of various stages and 97 control subjects. Disease severity was assessed using the UPDRS and Hoehn and Yahr staging. The LBP level between the PD and control groups was compared using analysis of covariance. Spearman correlation was used to explore the relationship between LBP level and disease severity.

**Results:** The mean LBP level in PD subjects (*n* = 94) was significantly different from control subjects (*n* = 95, *p* = 0.018). In PD subjects, we did not find a correlation between mean LBP level and disease severity.

**Conclusions:** Our data suggests that LBP is one GI biomarker related to LPS induced neurotoxicity. However, there was significant variability in LBP levels within the PD and control groups, limiting its utility as a stand-alone biomarker. This study supports the role of LPS induced neurotoxicity in PD and further exploration of this pathway may be useful in developing sensitive and specific biomarkers for PD.

## Introduction

The cause of Parkinson's disease (PD) is currently unknown. Both environmental and genetic factors have been identified that contribute to PD pathogenesis. The pathology of PD has been found throughout the entire nervous system including the central, peripheral, and enteric nervous system (Braak and Del Tredici, 2008).

Environmental factors can trigger an inflammatory cascade in genetically susceptible individuals resulting in neurodegeneration. In fact, activated microglia and increased proinflammatory cytokines (TNF, IL-1B, IL-6, iNOS) have been reported in the CSF (Mogi et al., 1994; McGeer et al., 2001) of PD subjects. There is a growing body of evidence that inflammation results in neurodegeneration in PD, rather than inflammation being a consequence of the disease (Côté et al., 2011, 2015). An inflammation-driven animal model of PD has been proposed based on lipopolysaccharide (LPS) induced neurotoxicity (Mogi et al., 1994). LPS is an endotoxin found on the outer membrane of gram negative bacteria and humans are exposed to LPS through the intestinal tract (Sharma and Nehru, 2015). The intestinal tract and thus the enteric nervous system may serve as a conduit to the central nervous system. It has been posited that the inflammatory process could gain access to the lower brainstem via the vagal nerve and then ascend through the basal mid- and forebrain until it reaches the cerebral cortex, producing various pre-motor and motor symptoms of PD along the way (Braak and Del Tredici, 2008). LPS may be one of the inflammatory triggers involved in this process.

Systemic exposure to bacterial endotoxin can be determined by measuring plasma LPS binding protein (LBP). Low levels of plasma LBP have been associated with increased gram negative bacterial exposure. LBP is an acute phase protein that binds to LPS to induce immune responses by presenting LPS to cell surface markers including CD14 and TLR4 (Muta and Takeshige, 2001). Ultimately, this may trigger monocyte responses resulting in secretion of inflammatory markers such as IL-6 (Thomas et al., 2002). A study of 9 patients with early PD (median Hoehn and Yahr stage 2) and age matched controls found that the PD subjects had a significantly lower mean level of plasma LBP compared to control subjects (Forsyth et al., 2011). Lower levels of plasma LBP, as shown in these PD subjects, have been associated with increased exposure to gram negative bacteria (Gutsmann et al., 2001; Minter et al., 2009) which supports the hypothesis that PD subjects have increased intestinal permeability to gram negative bacteria and bacterial products.

The aim of the research plan was to evaluate LBP as a potential biomarker for PD across a spectrum of disease severity. We examined whether reductions in LBP correlate with measures of PD severity.

## Materials

Blood (6 cc) was collected using normal aseptic techniques and samples were initially kept on ice. Plasma was separated from the blood within 20 min after blood sampling by centrifugation (1500 × g at 4°C for 15 min). The plasma was subsequently transferred to a fresh polypropylene tube and stored at −70°C. Samples were collected over a 6 month period. Once all of the required samples were collected, they were thawed and used within 24 h of thawing. LBP levels were measured using the Hycult biotech Human LBP Elisa kit (HK315) (Hycult Biotech, 2013). and all samples were processed concurrently to maintain consistency of the measurements. All plates were processed simultaneously to limit batch effects. Samples were run in duplicate. Briefly, samples and standards were incubated in microtiter wells coated with antibodies recognizing human LBP. Biotinylated tracer antibody was bound to captured human LBP. Streptavidin-peroxidase conjugate was bound to the biotinylated tracer antibody. Streptavidin-peroxidase conjugate react with the substrate, tetramethylbenzidine (TMB). The enzyme reaction was stopped by the addition of oxalic acid. The absorbance at 450 nm was measured with a spectrophotometer. A standard curve was obtained by plotting the absorbance (linear) vs. the corresponding concentrations of the human LBP standards (log). The human LBP concentration of samples, which were run concurrently with the standards, was determined from the standard curve.

## Methods

This observational study was cross-sectional in design and subjects were enrolled from the Rush Movement Disorders clinic. The study proposal and consent were approved by the Institutional Review Board of Rush University. All participants (or their legal representatives) provided written, informed consent to participation. One-hundred PD subjects and 100 controls were enrolled from June 2013 to January 2014. All PD patients met the UK Parkinson's disease society brain bank clinical diagnostic criteria (Hughes et al., 1992) and the diagnosis of PD was confirmed by G.P. after personal interview, medical history, physical examination, and chart review. Clinical and demographic data included age at time of enrollment, gender, ethnicity, disease severity, gastrointestinal symptom severity, and LBP level. Exclusion criteria for control groups: history of GI malignancy, undergoing treatment with medications that may induce parkinsonism (metoclopramide, typical, or atypical antipsychotic agents), known diagnosis of inflammatory bowel disease, presence of symptomatic functional GI disease that significantly impairs intestinal motility such as scleroderma or use of GI motility drugs, presence of an acute illness requiring immediate hospitalization, prior diagnosis of short bowel syndrome or severe malnutrition. Subjects were not excluded for laxative use.

Disease severity was assessed by G.P. for each patient at the time of data and blood collection. Disease severity was assessed with the Unified Parkinson's Disease Rating Scale (UPDRS), Hoehn and Yahr (H&Y) staging, and Schwab and England scale (S&E) in the “ON” medication state.

The current status of gastrointestinal symptoms was determined using a self-report survey with a bowel habits questionnaire, Gastrointestinal Symptom and Severity Rating Checklist (GISSC), administered to the patient at the routine visit. This questionnaire includes a validated structured questionnaire that is used to assess bowel habits (Forsyth et al., 2011) and the Bristol stool consistency score card (Basseri et al., 2011). Higher questionnaire scores indicate more prominent gastrointestinal symptoms.

G^*^Power version 3.1 was used determine the power based on the difference between two independent means derived from the data by Forsyth et al. (2011) We assumed a mean of 84291 ng/ml ± 31380 for the control group and a detectable difference of at least 15% (12644 ng/ml). With an a priori analysis, and a two tailed test (α = 0.05, power of 80%) the required sample size was 97 subjects per group. We planned to recruit 100 subjects per group which would provide >80% power and likely even higher given the large standard deviation.

SPSS version 16.0 was used for additional statistical analysis. Descriptive analysis was performed on all variables and their distribution assessed. LBP levels were not normally distributed. Nonparametric analyses with the Mann-Whitney test was used to compare mean LBP levels in the PD and control groups. LBP levels were transformed to z-scores and outliers that were ≥3 standard deviations were excluded and repeat analyses were performed. An analysis of covariance (ANCOVA) was conducted adjusting for covariates. Correlations were calculated using Spearman's rho (r), where the accuracy of the analysis was assessed by the area under the curve (AUC) of the receiver operating characteristic (ROC) curve. Cutoff values of LBP were calculated using sensitivity and specificity values of 80%.

## Results

Of the 100 PD subjects enrolled, samples were successfully processed in 94 subjects. Ninety-nine of the 100 control samples were adequately processed. Demographic data are listed in Table 1. PD subjects were significantly older than controls subjects (*p* = 0.001). Also, there was a significant difference between the numbers of men and women in the two groups (*p* < 0.001). Mean LBP level was significantly lower in the PD group (*n* = 94) compared with the control group (*n* = 99) (9344 ± 6694 vs. 12330 ± 8912 respectively, *U* = 3573.5, *p* = 0.005) (Table 1).

**Table 1:** **Demographics**.

	**PD (*n* = 94)**	**Control (*n* = 99)**	***P***
Age, mean	67.7 ± 10.5	62.6 ± 10.7	0.001
**GENDER**, ***n*** **(%)**			
F M	37(39.4%) 57(60.6%)	70(70.7%) 29(29.3%)	< 0.001
Total UPDRS	42.8 ± 14.1	−	
HY stage	2.3 ± 0.64	−	
Schwab and England	81.0 ± 12.3	−	

*Values are mean ±SD. PD, Parkinson's disease; GI, Gastrointestinal; LBP, lipopolysaccharide binding protein; UPDRS, Unified Parkinson's disease Rating Scale*.

**Figure 1:**
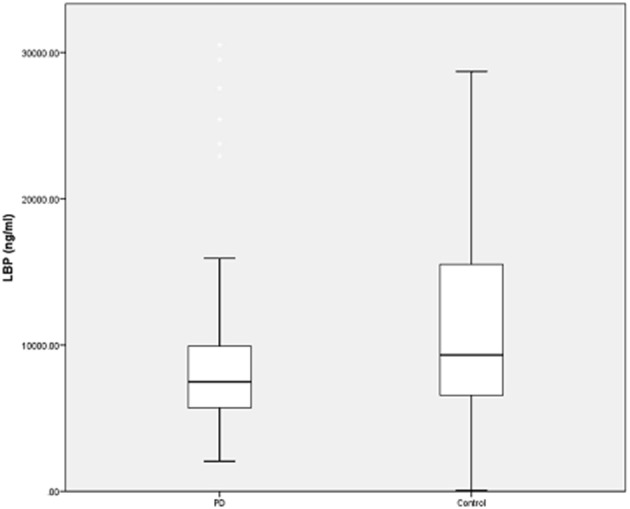
**LBP levels in PD and control subjects**. Plasma LBP is significantly lower in PD patients. However, there remains significant overlap between the two groups and wide variability regarding the assay in both groups. Data are presented as means (ng/ml).

To determine possible outliers, z-scores were obtained and values that were ≥3 standard deviations were removed. Four outliers were excluded in this fashion (all in the control group) and additional analyses were performed. There remained a significant difference in LBP levels between the PD group (*n* = 94, LBP 9344 ± 6694 ng/ml) and the control group (*n* = 95, 11280 ± 7422 ng/ml) (*U* = 3573.5, *p* = 0.018) (Figure 1). There was also a significant difference between GISSC scores and bowel movements per week in the PD group compared with the control group (Table 2).

**Table 2:** **Comparison of LBP level, GISSC scores, bowel movements per week**.

	**PD (*n* = 94)**	**Control (*n* = 95)**	***U***	***p***
LBP level (ng/ml)	9344 ± 6694	11280 ± 7422	3573.5	0.018
GISSC, Mean	35.9 ± 38.5	15.1 ± 20.4	2879.0	< 0.001
Bowel movements per week, mean	6.8 ± 5.0	8.6 ± 4.6	3190.5	< 0.001

*Values are mean ±SD. PD, Parkinson's disease; GI, Gastrointestinal; LBP, lipopolysaccharide binding protein; UPDRS, Unified Parkinson's disease Rating Scale. U, Mann-Whitney U-score*.

Given that age and gender were significantly different between the PD and control groups, these variables were used as covariates in an ANCOVA model. The difference in LBP levels between PD and control groups remained significant (*p* = 0.03). The covariates age (*p* = 0.196) and gender (*p* = 0.704) were not significant predictors of LBP level.

In the PD group, there was no correlation between mean LBP level and UDPRS score (*r* = −0.022, *p* = 0.830), HY stage (*r* = −0.037, *p* = 0.727), or GISSC score (*r* = −0.087, *p* = 0.404). There was no correlation between LBP level and SE score (*r* = −0.038, *p* = 0.716) or bowel movements per week (*r* = −0.101, *p* = 0.332). When both PD groups and control groups were taken together, there was no correlation between mean LBP level and GISSC score (*r* = 0.048, *p* = 0.508) or bowel movements per week (*r* = −0.014, *p* = 0.849).

**Figure 2:**
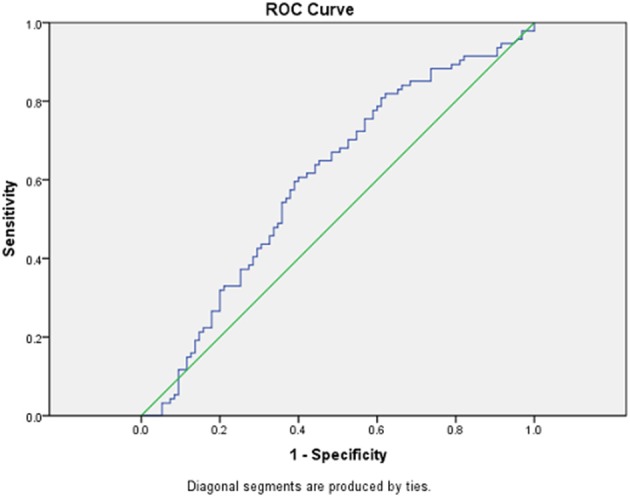
**ROC curve for PD vs. control group**. Receiver operating curve (ROC) for LBP levels between 94 PD subjects and 95 control subjects.

We performed a sensitivity analysis and constructed an ROC curve to determine the utility of this test. With the sample of 94 PD subjects and 95 control subjects, an AUC of 0.60 (95% CI = 0.518–0.681) was obtained (Figure 2).

## Discussion

Our data suggests that lipopolysaccharide binding protein (LBP) is one GI biomarker related to LPS induced neurotoxicity that is significantly different between PD and control subjects. However, there was significant variability in LBP levels within each group and significant overlap between the PD and control groups that was much greater than our previous report (Forsyth et al., 2011). This is likely because we recruited a broader spectrum of PD subjects, whereas the report by Forsyth et al. (2011) only recruited subjects with early PD. Our results also show that LBP has a poor predictive value with an AUC of 0.62. This limits the utility of LBP as a stand-alone biomarker. Ultimately LBP seems to be a marker of immune dysfunction and inflammation (Froon et al., 1995), though it has been linked to other systemic disorders including obesity (Huang et al., 2015) and atherosclerosis (Serrano et al., 2013), which may further account for its wide variability. However, additional markers related to LPS induced neurotoxicity may warrant exploration. These GI markers include: (1) zonulin and intestinal fatty acid binding protein (IFABP): markers of intestinal permeability and integrity; (2) endotoxin and serum CD14 (sCD14): markers of gram negative bacterial inflammation; (3) lipoteichoic acid (LTA): a marker of gram positive bacteria (Forsyth et al., 2011). If LPS truly is involved in PD pathogenesis, zonulin, IFABP, LPS, and sCD14 would be expected to be abnormal in PD subjects compared with controls. Further, there would be no difference in LTA levels between PD and control groups since gram positive bacteria are not involved in LPS inflammatory response. Thus, understanding the pathologic effects of LPS may be useful in developing a panel of biomarkers which are both sensitive and specific to PD subjects.

We did not find a correlation between LBP level and disease severity. PD is a heterogenous disease and GI inflammation may not be the pathologic culprit in all subjects afflicted by the disease. Thus, additional studies are needed to parse out subjects who may have GI pathology as the underlying cause which may further increase the utility of GI biomarker testing. Another explanation for the lack of LBP correlation with disease severity may relate to the role of LPS as an inflammatory trigger. Since LPS is considered an acute phase reactant (Froon et al., 1995), it may be abnormal only in the early stages of PD and may subsequently normalize as the disease progresses. Additional studies are needed to explore this possibility.

Study limitations include a small representation of non-Caucasians and a significantly higher number of women recruited to the control group. This is not surprising since PD is more common in men (Wooten et al., 2004) and the control subjects were often spouses of PD subjects who were recruited to participate in the study. Using ANCOVA, when we adjusted for gender, our results remained statistically significant. Also, our control subjects did not have a neurological examination performed to exclude Parkinsonism. Since PD is associated with a potentially long pre-motor phase (Pont-Sunyer et al., 2015) it is possible that some of the control subjects may have had early or pre-motor Parkinsonism. Lastly, it is unknown whether LBP is specific to PD or whether LBP plays a role in atypical Parkinsonism as well (ex. MSA). This is an area where further research is needed.

Our PD subjects were also significantly older than controls and the male predominance of PD with younger spouses acting as controls may play a role in this as well. LBP levels in young, healthy controls have been found to be lower than in the older healthy population (Stehle et al., 2012). However, our analyses remained statistically significant when we controlled for age. Furthermore, our control population was younger than the PD population so they would be expected to have lower levels of LBP compared with age matched controls, which would potentially make the difference between the PD and controls groups even larger had we used age matched controls.

In conclusion, LPS is an endotoxin that has been suggested to play a role in the pathogenesis of PD. Further studies of LPS, LBP, and other markers of GI inflammation are needed to develop a set of reliable biomarkers that are useful in distinguishing between PD and control subjects.

## Author Contributions

GP: Drafting/revising the manuscript for content, including medical writing for content, Analysis or interpretation of data. MS: Analysis and interpretation of data. CF: Study concept and design. BO: Analysis and interpretation of data. AK: Study concept and design. KS: Drafting/revising the manuscript for content, including medical writing for content, Study concept, or design, Analysis or interpretation of data.

## Funding

This promising research has been funded by philanthropic support from the family of Leslie Nan Burridge, Phillip Garoon, Khurram and Sameera Hussain and the Parkinson's Disease Foundation, Department of Neurological Sciences, Rush University.

### Conflict of interest statement

Ali Keshavarzian is part owner of Nutrabiotix Inc., that develops prebiotics to treat dysbiosis and has a patent for one prebiotic. The author has not had any revenue from the Nutrabiotix yet and the only source of revenue for the company so far is an NIH grant. The other authors declare that the research was conducted in the absence of any commercial or financial relationships that could be construed as a potential conflict of interest.
